# Comparison of laterally condensed, vertically compacted thermoplasticized, cold free-flow GP obturations – A volumetric analysis using spiral CT

**DOI:** 10.4103/0972-0707.58334

**Published:** 2009

**Authors:** Deivanayagam Kandaswamy, Nagendrababu Venkateshbabu, Reddy Gopi Krishna, Rosaline Hannah, Ganesh Arathi, Riaz Roohi

**Affiliations:** Department of Conservative Dentistry and Endodontics, Sri Ramachandra Dental College, Porur, Chennai, Tamil Nadu, India

**Keywords:** Cold, free-flow obturation technique, lateral condensation, spiral computed tomography, thermoplasticized gutta-percha, vertical condensation

## Abstract

**Aim/Objective:**

To compare the laterally condensed gutta-percha, vertically compacted thermoplastized gutta-percha (E and Q Plus system) and cold free-flow gutta–percha (GuttaFlow). This is a volumetric analysis using spiral CT, an *in vitro* study.

**Materials and Methods:**

Access cavities were prepared in 60 single rooted anterior teeth; cleaning and shaping was done and obturated with three of the different techniques: group A: cold lateral; group B: vertically compacted thermoplasticized and group C: cold free-flow obturation techniques. Volume analysis was done using spiral computed tomography (CT). The percentage difference was calculated and statistically analyzed using one-way ANOVA and post hoc multiple comparison Tukey HSD tests.

**Results:**

There were statistical significant differences between group A (0.183cm^3^) and group B (0.136cm^3^); group A (0.183cm^3^) and group C (0.128cm^3^). But there was no statistical significance between group B (0.136cm^3^) and group C (0.128cm^3^).

**Conclusion:**

Within the limitations of this *in vitro* study it can be concluded that cold free-flow obturation technique showed the highest volume of obturation, followed by the vertically condensed thermoplasticized technique. The least volume of obturation was observed in cold lateral condensation technique.

## INTRODUCTION

The objective of endodontic obturation is to provide a complete seal along the length of the root canal system, thereby ensuring the healing and sustained health of the peri-radicular tissue.[1] The root canal filling material should provide a barrier that prevents bacteria from the oral cavity from travelling down the root canal.[2]

Gutta–percha (GP) is the most commonly used root canal obturation material.[3] It is compressible, inert, dimensionally stable, tissue tolerant, radiopaque, and becomes plastic when heated.[3] Its physical properties have made possible several obturation techniques. The cold lateral condensation of GP is one of the most commonly used techniques in endodontics. However, its ability to replicate the internal surface of the root canal has been questioned. Voids, spreader tracts, incomplete fusion of the GP cones, and lack of surface adaptation have been reported.[4]

The thermoplasticized injectable obturation techniques were introduced to improve the homogenicity and surface adaptation of GP. Budd *et al*. compared the quality of obturation using high and low temperature thermoplastic injectable GP techniques with lateral condensation and proved both the thermoplasticized injectable techniques were significantly better than lateral condensation.[5] Overfilling occurred 75% of the time with vertical condensation of thermoplasticized GP.[6] In order to overcome the flaws of apical extrusion and shrinkage in the thermomplasticized condensation, cold, free-flow obturation technique was introduced.

In 2004, Coltene/Whaledent Inc (Cuyahoga Falls, OH) introduced a cold, flowable, self-curing obturation material for root canals that combines GP and sealer into one injectable system. GuttaFlow contains GP in particle form combined with a polydimethylsiloxane – based sealer. GuttaFlow is available in a capsule and can be injected directly in the canal. It is used in combination with a master GP cone and does not require any form of manual compaction for placement.[7] According to the manufacturer, GuttaFlow has excellent flow properties because its viscosity diminishes under shear stress (thixotropicity).[8] The material is believed to flow into lateral canals and completely fill the space between the root canal and the master cone. In addition, because no heat is used with placement of the material, no shrinkage is believed to occur, and the manufacturer reports that the material expands 0.2% upon curing.[9]

In a previous study of volumetric shrinkage, the teeth were sectioned longitudinally and photos taken, which were analyzed with digital image processing to measure the surface area covered with the obturation material.[10] In our study, volume analysis was done with spiral CT. Volume analysis gives a more accurate measure than surface area measurement.

With spiral CT, three-dimensional volume measurements are possible without sectioning the specimens and thus avoiding the loss of material during sectioning. Nandini *et al*. used volumetric analysis by spiral CT to determine the removal efficiency of calcium hydroxide intracanal medicament with two calcium chelators.[11]

The aim of our study was to compare the laterally condensed GP, vertically compacted thermoplastized GP (E and Q Plus system) and cold free-flow GP (GuttaFlow); a volumetric analysis using spiral CT, an *in vitro* study.

## MATERIALS AND METHODS

Sixty extracted single rooted anterior teeth were selected. The teeth with fractures, cracks, or any other defects were excluded from the study. Teeth were stored in normal saline solution which was changed daily till sufficient number of teeth was collected. Access was prepared and the root canals were subjected to chemomechanical preparation with the step-back technique using K-files (Maillefer, Ballaiges, Switzerland). The master apical file was standardized to three times the size of the initial apical file and 5.25% NaOCl was used as an irrigant after each instrument. Recapitulation with smaller size files was done during chemomechanical preparation. The teeth were stored in normal saline in airtight bottles in between procedures. The teeth were divided into three groups of 20 teeth each and mounted on a plastic stand using modeling wax to take spiral CT. After CT imaging, the volume of the root canal in each tooth was estimated using Siemens Emotion Duo model of Spiral CT with the aid of Syngo software.

Group 1: Cold lateral compaction technique: A standardized GP (Maillefer, Ballaiges, Switzerland) master cone was fitted with tugback 0.5mm from the open apical foramen. AH plus sealer (Dentsply Detray, Konstanz, Germany) was applied to the root canal wall using a finger spreader(Maillefer, Ballaiges, Switzerland) with a counter clockwise rotation. The apical part of the master cone was coated with sealer and introduced slowly into the root canal until the working length was reached. Lateral compaction was done using standardized finger spreaders and medium–fine accessory GP cones (Maillefer, Ballaiges, Switzerland) coated with sealers were used.

Group 2: Vertical thermoplasticized compaction technique: Canals were filled using the E and Q Plus system (Meta Dental Corp.) according to the manufacturer's instructions. A heating tip in the pen-grip handpiece was selected to fit in the root canal without binding, 4-mm short of the working length. A standardized GP cone (Dentsply Maillefer) exhibiting, a “short, crisp” tug-back sensation at 0.5 mm short of the working length was chosen. A thin coat of sealer was applied to the root canal walls to the approximate depth of the master cone using a size 35 file. Then, the master cone was lightly coated with a sealer at its apical one-third and placed into the root canal. The heating tip was activated to a setting of 200°C, and the coronal excess of the GP cone was seared off at the orifice. The activated tip was then inserted in a slow, steady motion into the canal to a depth 4-mm short of the working length and was maintained there for three to four seconds. The tip was then allowed to cool for 10 seconds and removed after a single burst of heat applied for about one second. The backfilling of the canal was achieved by injection of thermoplasticized GP by using the E and Q gun until the canal was completely filled with GP. This technique was similar to the continuous wave of condensation technique.[12]

Group 3: Cold free- flow compaction technique: Following the manufacturer's instructions, the GuttaFlow plastic insertion tip was placed into the canal to a depth at which the tip no longer advanced. The GuttaFlow filling depth starting point was established 3 mm short of this length, and the tip was bent to serve as reference for appropriate placement during obturation. The GuttaFlow capsule was activated and triturated for 30 seconds, the plastic tip was attached to the capsule, and a small amount of material was dispensed onto a pad. The color of the GuttaFlow was compared with the manufacturer's color scale to ensure that the material was mixed appropriately. The tip was inserted into the canal to the filling depth starting point, and material was dispensed until it could be seen moving up the canal around the tip. A standardized GP master cone (Maillefer, Ballaiges, Switzerland) was coated with GuttaFlow and inserted into the canal to the WL. The cone was gently pulled upward 2 to 3 mm, twisted twice, and reseated to the WL.

The canal was backfilled with GuttaFlow by placing the plastic insertion tip next to the master point to a depth at which the tip was neither forced nor bound the canal wall. A heated hand instrument was used to remove the coronal extension of the master point such that the obturation material was flush with the canal orifice. The material was allowed to self-cure for 50 minutes. A second spiral CT was done and the volume of obturation in each tooth was estimated as before. The volume of obturation was calculated as {(a-b)×100/a}, where ‘a’ was the volume of the root canal after chemo-mechanical preparation and ‘b’ was the volume of the root canal after obturation with the three different techniques.

### Statistical analysis

The data was statistically analyzed using SPSS version 10.0.5 software and tested using one-way ANOVA (*P* < 0.001). Intergroup comparisons were done using post hoc multiple comparisons Tukey HSD tests (*P* < 0.001) assuming unequal variance.

## RESULT

**Figure 1:**
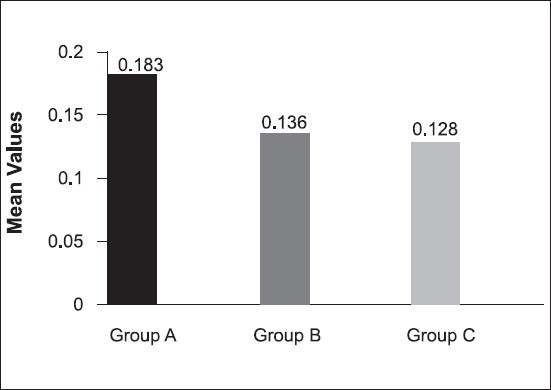
Volume of voids seen in the three groups

The results are summarized in Figure 1. There was statistical significant difference between group A (0.183cm^3^) and group B(0.136cm^3^); group A(0.183cm^3^) and group C(0.128cm^3^). But there was no statistical significance between group B (0.136cm^3^) and group C (0.128cm^3^).

## DISCUSSION

The root canal is a complex system with many surface irregularities, fins, accessory and lateral canals, and isthmuses.[13] To seal this system, the filling material must adapt to all portions of the root canal. Incomplete obturation of the canal system may result in failure of the endodontic treatment.[14] Root canal filling materials are intended to prevent micro-organisms and toxins in the canal from passing along the root canal space into the periradicular tissues.[1]

In a fluid-filtration study,[15] GuttaFlow with single-cone provided coronal seal which was inferior to that of AH Plus. The authors recommended placement of accessory cones to reduce sealer thickness coronally.[15] In another fluid filtration study,[12] GuttaFlow also showed inferior sealing than AH Plus in combination with different obturation techniques. When a lentulo spiral and no gutta-percha cones were used, GuttaFlow provided a similar seal to that of AH Plus. Ozok *et al*.[8] reported that GuttaFlow had the highest amount of leakage because the matrix of this thixotropic sealer might flow under the pressure applied by the inserted gutta-percha cones, leaving only the gutta-percha particles between the cones and the dentin wall.

Elayouti *et al*.[16] evaluated the presence and area of the voids within GuttaFlow fillings and between the sealer and the root canal wall at five apical-coronal levels. They proved that the mean area of the voids was the lowest in GuttaFlow group, the frequency of the voids was significantly higher than the conventional cold lateral and warm vertical compaction of GP in combination with AH Plus. The significantly high frequency of the voids at all measurement levels in the GuttaFlow group, although smaller in area, indicates an increased possibility of communication between these voids and the apical and coronal ends of the root canal filling. They also observed that in this group the voids were almost always within the sealer itself but not at the sealer-dentin interface. Nearly all voids in the GuttaFlow were enclosed within the core of the filling material, thus the adaptation to root canal was almost complete. The porosity of the sealer might be a result of using a lentulo spiral to place the highly viscous gutta-percha paste into the canal or simply because of the manufacturing process.

Kontakiotis *et al*.[17] reported that when comparing the contact angles of GuttaFlow, RoekoSeal (Roeko, Langenau, Germany), AH26 (Dentsply De Trey GmbH, Konstanz, Germany), and Roth's 801, AH26 and Roth's 801 showed significantly smaller contact angles that the silicone – based sealers. Such a finding implies decreased wettability of GuttaFlow compared with conventional sealers.

Numerous *in vitro* investigations have evaluated obturation techniques by comparing different variables such as length of fill,[18] defect replication,[19] and GP density.[20] In the current study, the focus was on the volume of obturation. Volume analysis was done with spiral CT. Volume analysis gives a more accurate measure than surface area measurement.[11] With spiral CT, three-dimensional volume measurements are possible without sectioning the specimens and thus avoiding the loss of material during sectioning.[11]

Nandini *et al*. used spiral CT for the volumetric analysis to assess the efficiency of two calcium chelators namely 17% EDTA solution and 10% citric acid combined with ultrasonic agitation, in the removal of calcium hydroxide placed as an intracanal medicaments.[11]

**Figure 2:**
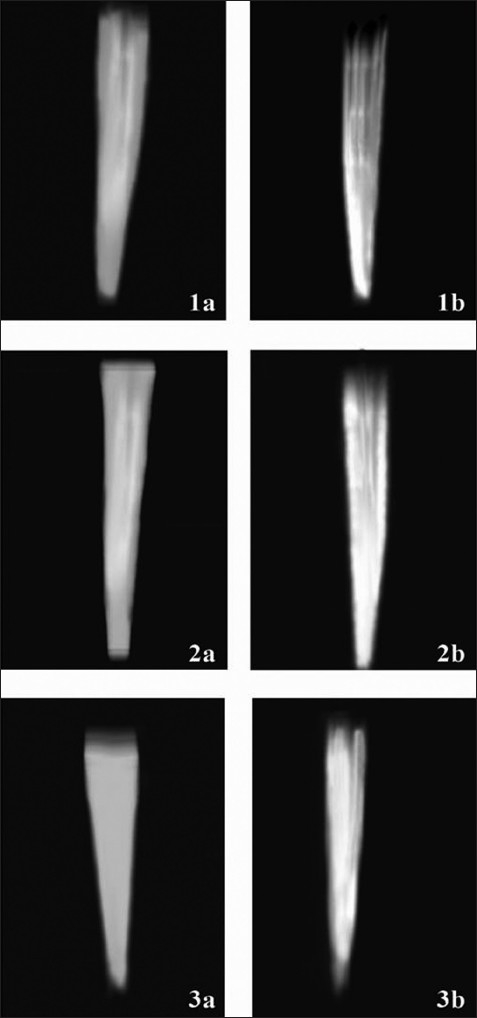
Group 1a, 2a and 3a, prepared canals. Group 1b, obturated with lateral condensation technique. Group 2b, obturated with vertically condensed thermoplasticized technique. Group 3b, obturated with cold, free-flow gutta-percha

In the current study, GuttaFlow showed highest volume of obturation in comparison to lateral condensation and vertically compacted thermoplasticized E and Q Plus System [Figure 2]. The reason for this could have been the better flow thereby increasing the wettability for GuttaFlow than the other two groups.

Lateral condensation group showed the least volume of obturation because this technique has shown to form a nonhomogenous mass of gutta-percha that poorly replicates the prepared root-canal space and does not adequately obdurate simulated lateral canals.[18] Additionally, lateral compaction technique leaves voids between cones[21] that often are not filled with sealer[22] and may provide a niche for bacteria to thrive.

## CONCLUSION

Under the limitations of this study, cold free-flow obturation showed the highest volume of obturation, followed by vertically compaction thermoplasticized technique; the least volume of obturation was observed in cold lateral compaction.
